# Short-Term Effects of Intra-Articular Hyaluronic Acid Administration in Patients with Temporomandibular Joint Disorders

**DOI:** 10.3390/jcm9061749

**Published:** 2020-06-05

**Authors:** Maciej Sikora, Barbara Czerwińska-Niezabitowska, Maciej Adam Chęciński, Marcin Sielski, Dariusz Chlubek

**Affiliations:** 1 Department of Maxillofacial Surgery, Hospital of the Ministry of Interior, Wojska Polskiego 51, 25-375 Kielce, Poland; sikora-maciej@wp.pl (M.S.); marcinsielski@gazeta.pl (M.S.); 2 Department of Biochemistry and Medical Chemistry, Pomeranian Medical University, Powstańców Wlkp. 72, 70-111 Szczecin, Poland; 3 Specialist Orthodontic Practice: Treatment of Masticatory Organ Dysfunction, Kujawska 19/41, 25-344 Kielce, Poland; baja@netcom.kielce.pl; 4 Department of Maxillofacial Surgery, University Clinical Hospital, Wincentego Witosa 26, 45-401 Opole, Poland; maciej@checinscy.pl

**Keywords:** hyaluronic acid, intra-articular administration, intra-articular injection, temporomandibular joint disorders, viscosupplementation

## Abstract

The study described in this paper was conducted to assess the short-term outcomes of intra-articular administration of hyaluronic acid in patients with symptoms of temporomandibular joint disorders. A group of 40 patients suffering from temporomandibular joint disorders underwent a series of hyaluronic acid intra-articular injections. Questionnaires and clinical examinations were conducted to assess stress exposure of the subjects and to evaluate short-term treatment outcomes, i.e., reducing joint and muscle pain and increasing the mobility of the mandible. A weak positive correlation between stress exposure and pain was observed. As a result of treatment, 61% of subjects revealed a total reduction of muscle pain, while joint pain completely resolved in 88% of patients. Mandibular mobility increased by 11%, 31%, 9%, and 11% regarding opening, protrusive, and lateral right and left movements, respectively. The study confirms the short-term effectiveness of intra-articular administration of hyaluronic acid on reducing joint and muscle pain in patients with articular disc displacement. The treatment positively affected the mobility of the mandible in all directions. The verification of late treatment effects of hyaluronic acid viscosupplementation requires the continuation of the research.

## 1. Introduction

Temporomandibular joints (TMJs) belong to the category of synovial joints in the human body, where joint surfaces are lubricated by the synovial fluid produced by the synovial membrane lining the joint capsule. The synovial fluid consists mainly of hyaluronic acid (HA)—an anionic, nonsulfated glycosaminoglycan and lubricin—a surface-active mucinous glycoprotein.

An appropriate concentration of HA in the synovial fluid is crucial for proper tissue elasticity and for reducing friction between the joint surfaces of the bone and the synovial disc. In this way, the viscoelastic properties of the synovial fluid protect the chondrocytes against mechanical damage caused by excessive pressure. HA is also responsible for suppressing stresses generated inside the joint when subjected to trauma. In addition, HA has antioxidant properties, reducing the amount of free radicals present in the joint cavity. HA also facilitates the penetration of nutrients from the blood through joint mucus into the joint cartilage. Finally, by interacting with collagen, HA forms a barrier against microorganisms and toxins.

Viscosupplementation, i.e., supplementation with a component of synovial fluid in the form of intra-articular injections, is one of the possible solutions to reduced HA levels in joint cavities in chronic inflammation, already successfully used in the treatment of knee osteoarthritis [1]. Importantly, a detailed summary of 24 reports on 6 HA-based preparations used in the viscosupplementation of temporomandibular joints showed no serious adverse events [2].

## 2. Aim of the Study

Temporomandibular disorders (TMD) and related pain are becoming an increasingly common social problem. Patients suffering from TMJ pain seek help from specialists in various fields of medicine, including prosthodontists, orthodontists, neurologists, and physiotherapists. Importantly, the effects of monotherapies are often insufficient due to the interdisciplinary nature of the problem. Given the unsatisfactory results of current medical solutions and the scarcity of research on viscosupplementation with HA (usually on small groups of patients), in this study, we decided to investigate the effects of this therapy in patients with TMD.

## 3. Materials and Methods

This study was conducted in accordance with the Declaration of Helsinki. All procedures involving human subjects were approved by the Ethics Committee of the Pomeranian Medical University in Szczecin (Approval No. KB-0012/230/11/18). Written informed consent was obtained from all subjects. 

The study included 40 adult patients with symptoms of masticatory system disorders, who qualified for intra-articular HA injections in accordance with the guidelines published by Escoda-Francoli et al. [3] and developed on the basis of a meta-analysis of research on viscosupplementation of the temporomandibular joint published between 1966 and 2008. By adopting these criteria, our study included patients who were diagnosed with (i) disc displacement without reduction, (ii) disc displacement with reduction, and (iii) degenerative joint diseases. The study included patients who had previously been excluded from known somatic diseases, whose coexistence may affect the assessment of TMJ [4]. It was also ensured that patients did not take medicines that could conceal TMJ symptoms.

All patients who qualified for the study complained of pain, which was diagnosed as associated with TMJ dysfunction. The therapeutic regimen proposed by Abouelhuda et al. [5] was adopted. Patients who had no pain relief in the subsequent stages of the therapeutic regimen were included in the study. These stages were (i) pharmacological treatment, (ii) physiotherapeutic procedures, and (iii) splint therapy. In cases of contraindications or the patient’s disagreement with any of the types of treatment, it was not implemented. In none of the cases prior to the study was any arthrocentesis or arthroscopy performed. During HA injection therapy, the patients were not treated with anything other than painkillers (paracetamol, tramadol). Thus, between HA injections, patients were not treated with night splint, arthrocentesis, or arthroscopy. Patients were strongly advised to use painkillers only when necessary. No regular analgesic pharmacotherapy was performed during the study. Prior to individual follow-up visits, patients were not allowed to take painkillers during periods when their use could affect the examination result.

The group of 40 patients consisted of 36 women and 4 men, aged 18–69 years (43 years on average). The duration of symptoms before inclusion in the study ranged from 1 month to 20 years (the median was 11 months). In addition, 95% of respondents reported exposure to psychological stresses of varying intensity. According to the protocol of the International Network for Orofacial Pain and Related Disorders Methodology [6], the appropriate questions from the questionnaire by Dworkin and LeResche [7] were translated into Polish and used to assess the intensity of stress.

The questions asked the patients concerning the indicators of stress—somatic and psychological sensations in the last month. According to Dworkin and LeResche [7], somatic sensation includes, among others, tightness in throat and stomach ache. Psychological indicators of stress include feelings of guilt, entrapment, or loneliness. The intensity of each of the 32 sensations was assessed on a five-point scale from 0 to 4. After summing up the points, the number of points is divided by the number of questions and the collective result of stress exposure for a given patient is determined. The results were interpreted as follows: 0–1—no stress, 1–2—minimal stress, 2–3—moderate stress, and 3–4—severe stress. The characteristics of the study group are presented in Table 1.

The study group was divided according to the diagnostic criteria for TMD (DC/TMD) proposed by Schiffman et al. in 2014 [8] (Table 2).

We also used another questionnaire developed by a team of two orthodontists, two maxillofacial surgeons, and two prosthetic surgeons to evaluate the objective and subjective symptoms of TMD. It consists of manual functional analysis of TMD according to Bumann et al. [9] and palpation of head and neck muscles according to the protocol used by Felicio Festa and described by Tecco et al. in 2011 [10]. For the purpose of this paper, we used the Polish version of the muscular system examination developed by Czerwińska-Niezabitowska and Kulesa-Mrowiecka [11]. These techniques were used to evaluate joint and muscle pain, characterize the mandibular pathway during opening, and determine the amplitudes of mandibular movements in 3 planes. A preliminary examination using our own questionnaire was performed immediately before the first HA injection. During the qualification of patients for the study, the TMJ pain treatment performed so far was taken into account, according to the therapeutic ladder proposed by Abouelhud et al. [5]. Its elements are, sequentially, pharmacotherapy, occlusal splint therapy, intra-articular injections, physiotherapy, arthrocentesis, arthroscopy, and open TMJ surgery. Our study excluded those patients who had received any of these therapies during the three months preceding the study, except for “adhoc” analgesic pharmacotherapy without anti-inflammatory components. We have also excluded from this study the patients with local contraindications to intra-articular injections, according to the results of the review by Soni et al. [12]. These are abscess, inflammation or tumor of the skin, connective tissue or bone of the puncture site, bleeding diathesis or acquired coagulopathies, blood infections, and malignant tumors. Patients were re-examined at the last visit, 5–7 weeks after the first injection, which gave two dated and fully completed questionnaires for each patient.

The number and frequency of intra-articular injections were based on the recommendations of other researchers. All publications known to the authors of this paper proposed a series of up to 5 intra-articular HA injections. These observations are consistent with the analysis of literature on the administration of drugs to the temporomandibular joint cavity conducted 10 years ago by Mountziaris et al. [13], who identified two most commonly used HA viscosupplementation schemes: 2 injections at 7–14-day intervals or 5 injections at 7-day intervals. In our study, we adopted the regimen of 5 intra-articular HA injections at intervals of 7 to 10 days. The duration of intervals between these injections depended on the availability of patients.

For the purpose of this study, a protocol was developed for each of the 5 visits. On the first one, subjective and physical examinations were carried out on the basis of a questionnaire to qualify the patient for intra-articular injections. Viscosupplementation with HA was performed on each of 5 visits. During the last (fifth) visit, the injection was followed by subjective and physical examinations based on the questionnaire. Those patients who did not come for the fifth appointment—and so were not surveyed twice—were not included in the study. This inclusion criterion was met by 40 patients.

Out of the group of 40 patients who were present at the first and fifth appointments, 27 did not miss any of the appointments and received 5 HA injections. Ten patients canceled one of the visits (visits number 2, 3, or 4) and were given four intra-articular injections. The remaining three patients were only present at three appointments, and so received only three HA injections. The total absenteeism rate was 8%. In most cases, it resulted from unexpected events or satisfactory effects of treatment, which reduced the motivation to attend all five procedures.

The puncture site was determined in accordance with the protocol proposed by O’Connor et al. [14]. After depressing the mandible, on the line joining the lateral canthus of the eye with the cutaneous point tragus, we established a point 10 mm forward from the latter, and then the puncture site was determined by descending 2 mm below the point, perpendicularly to the described line. This location of the puncture site guaranteed the HA injection to the upper compartment of the joint. The skin was disinfected each time with a propanol-based preparation (Kodan Tinktur Forte) and the injection was performed when it was completely dry. No local anesthesia was used. All the injections were performed by the same maxillofacial surgeon. HA was administered at a dose of 0.4 mL of 2% hyaluronic acid (Synocrom) to one joint. In 19 patients, unilateral viscosupplementation was performed; in 21 patients, a bilateral injection was performed due to bilateral ailments.

The pain was determined on the basis of a clinical trial. Its presence was recorded for each of the patients in the preliminary and final questionnaires. Then, according to the protocol proposed by Skeie et al. [15], the pain was divided into muscular pain and joint pain, using a simple YES/NO questionnaire and palpation of head and neck muscles. Joint pain was assessed during the examination of the joint surface, joint capsule, and ligaments. This test was carried out in accordance with the manual functional analysis of masticatory system disorders. The joint surface was examined by dynamic protrusion compression and dynamic medial and lateral translations. The examination of the joint capsule and ligaments consisted of passive compression, stretching, and translation tests.

The opening pathway was evaluated in the preliminary test and after viscosupplementation. It is shown in a simplified Farrar’s diagram according to the protocol presented by Gorzałek and Kulesa-Mrowiecka [16]. In our study, anomalies of the opening pathway of less than 4 mm, called deviation by Okeson and Grocholewicz [17], were classified as an S-shaped mandibular opening pathway. Anomalies greater than or equal to 4 mm—permanent alteration, according to Okeson and Grocholewicz [17]—were described as deflection.

## 4. Results

In the study group, muscle pains were present in 36 patients before viscosupplementation and 14 patients after the treatment, which means a 61% effectiveness of the therapy in this regard (i.e., in 22 patients). Joint pain was present in 25 patients before the HA injections. At the end of the therapy, only 3 patients complained about this type of pain—it was not reported by 22 out of 25 (88%) patients. These data are presented in Table 3.

In 23 patients, muscle and joint pain coexisted before injection. The presence of both types of ailments after viscosupplementation was found in one patient. In 22 out of 23 people (96%), at least one of the types of pain disappeared. A person in whom both muscular and joint pain were present after the end of HA therapy was a 34-year-old woman who had reported the presence of pain in the right temporomandibular joint for about a month before the start of HA injections. She had not been treated previously for temporomandibular joint ailments. That patient reported no concurrent diseases, and on the basis of a questionnaire, we determined exposure only to moderate stress (approximately 2.5 points on a scale of 0–4). During the clinical examination, the patient was diagnosed with a dislocation of the joint disc with reduction, characterized by clearly limited mobility of the mandible: the opening amplitude was 32 mm, the protrusive and lateral movements were about 2 mm. HA viscosupplementation did not improve mandible mobility. The patient was qualified for further diagnostics, manual therapy, and possible treatment with the use of a repositioning splint.

The analysis of diagrams showing the movement of the mandible in the coronal plane allowed us to assess the influence of HA viscosupplementation on the opening pathway. In 5 patients, the anomalies of mandibular movement in the coronal plane did not exceed 4 mm. During the initial examination, the remaining 35 patients had pathological opening pathways (88% of the study group). Of these 35 subjects, an S-shaped opening pathway occurred in 26 patients and deflection in 9 patients. After completion of the intra-articular HA injection therapy, the mandible mobility in the frontal plane was normalized in 22 out of 35 patients (63%). Out of 26 patients with the S-shaped pathway, 16 (62%) patients showed satisfactory improvement at the end of treatment, and the remaining 10 (38%) patients maintained an alteration of at least 4 mm. In the group of 9 persons diagnosed with deflection, the therapy resulted in 6 (67%) patients obtaining a normal opening pathway during the final examination, while the remaining 3 patients (33%) showed no significant improvement.

**Figure 1:**
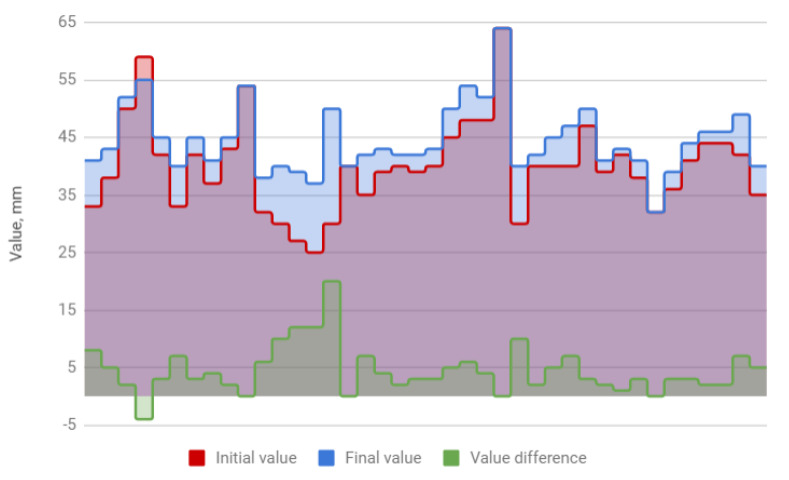
Mandible depression (columns represent values relevant to every patient).

**Figure 2:**
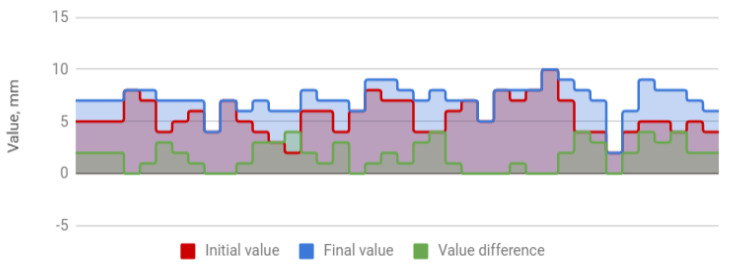
Mandible protrusion (columns represent values relevant to every patient).

**Figure 3:**
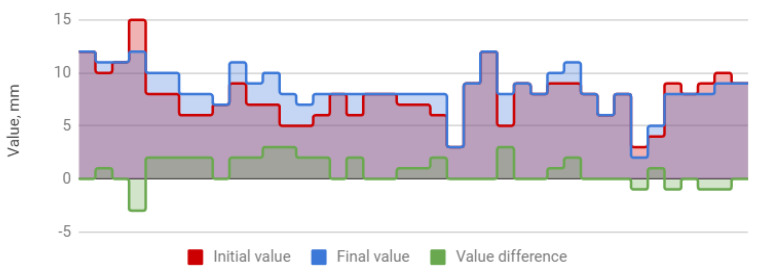
Right lateral movement of the mandible (columns represent values relevant to every patient).

**Figure 4:**
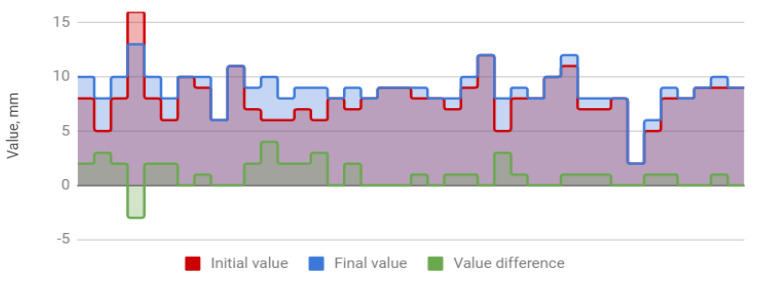
Left lateral movement of the mandible (columns represent values relevant to every patient).

The treatment resulted in an increase in the amplitude of mandibular movements in all planes. The mean opening movement in the study group was 40.1 mm. After HA injections, it increased by 4.5 to 44.6 mm, i.e., by 11%. The applied therapy had the greatest influence on the protrusion, which increased from 5.4 to 7.2 mm (by 31%). The range of lateral movement was 7.8 mm to the right and 8.0 mm to the left before the start of HA injections. It improved by 0.7 (by 9%) and 0.9 mm (by 11%), respectively, reaching 8.5 and 8.9 mm. Negative correlations were observed between the differences in the initial and final amplitudes and the initial amplitudes of individual extreme movements of the mandible. They indicate a weaker therapeutic effect of HA injections in cases of high initial mandibular mobility. The data discussed are presented in Table 4 and Figure 1, Figure 2, Figure 3 and Figure 4.

A correlation was also observed between the presence of pain before treatment with HA and the severity of stress experienced by patients. For the purpose of this report, pain was rated on a scale from 0 to 2, where 0 meant no pain, 1—muscle or joint pain, and 2—concurrent muscle and joint pain. Exposure to stress was determined on the previously mentioned scale from 0 to 4, resulting from an interview based on the questionnaire by Dworkin and LeResche [7]. The patients’ experiences from the period of 30 days preceding the initial examination were taken into account. Pearson’s correlation coefficient for stress and pain was 0.3, indicating a clear tendency for pain to increase in exposure to stress.

## 5. Discussion

As indicated by Panek and Maślanka [18], as well as by Kurpiel and Kostrzewa-Janicka [19], the terminology and classification of pathologies concerning temporomandibular joints have significantly evolved since the first written reports on the subject, i.e., since 1920. Despite the fact that the authors of various divisions were convinced about the relevance of their classifications, each of them was eventually replaced by a new one. An ideal classification should take into account anatomical and functional aspects, be clinically useful, support the physician in making therapeutic decisions, and at the same time, be concise and simple enough to be used in everyday practice. Among the many divisions, the closest to this ideal are the International Classification of Orofacial Pain (ICOP) [20] and DC/TMD [8]. As the study was conducted prior to the publication of the ICOP, we followed the guidelines proposed by Schiffman et al. under the DC/TMD protocols.

Painful ailments resulting from masticatory system disorders have a very complex nature. Harper et al. [21] attempted to analyze the mechanisms responsible for the pain associated with masticatory system disorders, distinguishing central and peripheral pain, and dividing the latter into nociceptive and neuropathic pain. In their conclusions, they emphasized that the cause of pain is no less important than the mechanism of its manifestation. Depending on the cause, the pain accompanying TMJ disorders can be divided into muscle and joint pain. Gorzałek and Kulesa-Mrowiecka [16] noted that isolated muscle pain restricts the lateral movements of the mandible without affecting its opening. This type of pain results from contractions and increased tension in the chewing muscles. According to Gorzałek and Kulesa-Mrowiecka [16], joint pain is associated with reduced opening and reduced amplitude of lateral movements. During the free opening, the mandible deviates towards the affected side.

Meticulous diagnostics of pain etiology allows us to take optimal therapeutic action. The method of treatment of muscle pain was proposed and examined by Pihut et al. [22]. These authors carried out intramuscular injections of botulinum toxin in 42 patients suffering from masseter muscle pain. The therapy resulted in a significant reduction in the incidence and severity of pain, which was also reflected in a reduction in the number of analgesics taken by patients. The intensity of pain of muscular origin was reduced in each of the patients.

In our study group, out of 36 people complaining of muscle pain, only 61% reported its total disappearance following HA viscosupplementation. This significantly lower therapeutic efficacy of intra-articular HA injections compared to intramuscular botulinum toxin administration is associated with the fact that intra-articular therapy only has an indirect effect on the masticatory muscle system. Similar effectiveness of HA viscosupplementation in the treatment of muscle pain was observed by Pihut et al. [23], where, out of 24 patients complaining of muscular pain, 71% stated that muscular pain subsided as a result of the therapy. Those authors compared the effectiveness of HA viscosupplementation with the results of intra-articular platelet-rich plasma (PRP) injections. Out of 25 patients treated with PRP, muscle pain disappeared in 68%. Similar results may indicate similar indirect efficacy of intra-articular injections with HA and PRP in the treatment of muscle pain.

With regard to joint pain, our results showed 88% effectiveness of HA viscosupplementation. It is difficult to put these results in perspective because, in the available literature, only Pihut et al. [23] identified joint pain as an isolated component of TMD, with pain relief experienced by 17 out of 22 patients (77%). Even given some differences in the method of establishing the presence of pain between Pihut et al. [23] and our study, HA viscosupplementation seems to be more effective in reducing joint pain than muscle pain.

In the treatment of pain resulting from TMD, intra-articular HA injections are used as a viable alternative to rinsing the joint cavity. However, the lack of a uniform protocol of management in TMD patients has resulted in various modifications of the treatment. These two methods are proposed to be used separately, and there are also recommendations for combining them. De Riu et al. [24] examined the effectiveness of intra-articular 2-mL HA administration preceded by rinsing the joint cavity with about 350 mL of saline. Pain on the VAS scale dropped from 8.26 to 2.03, which means 75% effectiveness of the therapy in 30 patients. However, that study did not distinguish between muscle and joint pain, in a typically synthetic approach that dominates the research on the effectiveness of TMD treatment with joint rinsing and viscosupplementation. The study by Gurung et al. [25] on the effectiveness of intra-articular HA injections as an additional procedure performed after rinsing the joint cavity is no exception. In the studied group of 10 patients, the pain intensity expressed on the VAS scale decreased from 5.9 to 1.3, i.e., by 78%, compared to a 56% pain reduction following joint rinsing only. In our study, a cumulative assessment of muscle and joint pain following HA therapy showed a 58% reduction in pain.

Widening the joint cavity by the mechanical displacement of the condylar processes downwards is also possible to achieve by conservative methods using various types of occlusal splints. However, their therapeutic effect is difficult to evaluate. Raphael and Marbach [26], in a study of 63 patients diagnosed with face and myofascial pain, did not observe any differences in the effectiveness of splint therapy compared to placebo. Pihut et al. [27] proposed intra-articular injections of rich platelet plasma in the case of ineffectiveness of occlusal splints used for joint disc displacement or increased chewing muscle tension. In the study conducted by those authors, good results were achieved by viscosupplementation of platelet-rich plasma in patients in whom splints had proved ineffective.

In our own study group, there were 6 patients whose complaints persisted as a result of previous therapies, and the effectiveness of viscosupplementation with HA in terms of pain relief was 83%. In this context, it is worth considering splint therapy as a preliminary treatment in sudden pain and unknown etiology. This approach was used by Shoush et al. [28], who compared the effectiveness of occlusal splints and therapeutic exercises. Those authors examined their effectiveness in terms of pain relief and normalization of mandibular opening amplitude in two 56-person groups of patients treated for 6 weeks. The first group used standard occlusal splints during the day. Patients from the second group took part in a series of 15-min exercises twice a week. The exercise session consisted of two parts. The first one included exercises proposed by Kijak et al. [29], similar to those developed by Gerry and described by Czerwińska-Niezabitowska and Kulesa-Mrowiecka [11]. The second part consisted of stretching the masseter and the medial pterygoid, according to Okeson’s protocol [30]. In the group using exercises, the efficacy of pain relief was improved by 9% and the amplitude normalization by 14%.

Akbulut et al. [31] showed much higher effectiveness of occlusal splint therapy, which resulted in pain relief and a significant increase in mandibular opening in 88% patients (*n* = 25). However, those authors admitted that during the first three months, they did not observe any significant effects of treatment with occlusal splints. It was only after 12 months of observation that the 88% success rate of the therapy was determined.

Akbulut et al. [31] defined the term “total healing” of TMD as simultaneous elimination of pain and normalization of opening amplitude. In their evaluation, however, they omitted an important indicator—the opening pathway. Our observations on the effect of HA viscosupplementation on the opening pathway in the coronal plane may be compared with the only study describing this parameter [23]. In our study, it improved in 63% of 35 patients, while Pihut et al. [23] described the normalization of this parameter in as many as 82% of 22 patients following intra-articular HA injections. These results clearly indicate a significant positive influence of HA viscosupplementation on the mandibular opening pathway. The observed differences may have resulted, among others, from different subjective methods of assessment of the opening pathway.

The method proposed by Kijak et al. [32]—based on a digital facial arch—offers a possibility to objectify the assessment of mandible mobility. The method includes a detailed analysis of the pathway of articular heads, which, in the future, may become a perfect complement to the imaging and manual functional analysis of TMD that we used in our examination. Using precise digital measurements, Kijak et al. [32] also determined the mean amplitudes of the mandibular opening. For the healthy group, the researchers calculated the average value of 45.6 mm. In 76 patients with diagnosed TMDs, the mean opening amplitude was 37.6 mm. In our group of patients, this value was 40.1 mm before the start of treatment. After the completion of the intra-articular therapy, HA increased to 44.6 mm, i.e., by 11%, which means that the final value was similar to the physiological value calculated by Kijak et al. [32].

**Figure 5:**
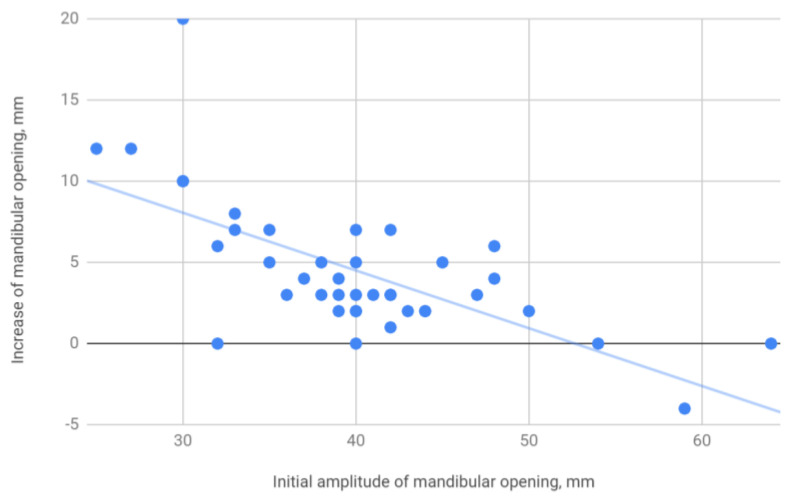
Initial amplitude of the mandibular opening and its increase following HA viscosupplementation.

Lewandowski [33] also showed a beneficial effect of HA viscosupplementation on the mandibular mobility assessed by the amplitude of its opening. However, the following years did not bring many studies on the impact of HA injections in this regard. Fonseca et al. [34] described a group of 10 cases of patients diagnosed with TMD. Mean amplitudes of mandibular opening before and after HA therapy increased from 30 and 37 mm, respectively, which allows us to determine the improvement of mandibular dislocation by 23%. This means that the HA viscosupplementation calculated in the analysis by Fonseca et al. [34] is more than twice as effective as in our study group. This discrepancy can be easily explained by the strong correlation between the difference between the initial and final amplitudes and the final amplitude of the mandibular opening. This correlation was calculated on the basis of data from our own study group and expressed as Pearson’s coefficient, *r* = −0.68. The significantly higher HA viscosupplementation efficiency for lower initial mandibular opening amplitudes in our study is shown in Figure 5.

The trend line shown in Figure 5 above can be represented by the following formula:

*y* = −0.4 × *x* + 18.7 mm (1)

where *x* is the initial amplitude of the mandibular opening and *y* is its increase following HA viscosupplementation. The mean final opening calculated using this formula would be 36.7 mm in the group of patients examined by Fonseca et al. [34], which is consistent with the actual posttreatment value of 37 mm presented in their study.

The improvement of mandibular mobility expressed as an increase in the amplitude of opening was also determined in groups treated with joint rinsing. In an analysis of 7 publications by the authors listed below, the relationship between the initial value of mandibular excitation and its increase in the course of therapy was also observed for joint cavity rinsing. It may be presented by the following formula:

*y* = −0.4 × *x* + 23.0 mm (2)

where *x* is the initial amplitude of mandibular opening, and *y* is an increase in the amplitude of the opening following joint lavage. In the discussed material, the final amplitudes of mandibular opening after joint cavity rinsing were, on average, as much as 5 mm higher than the expected results of treatment of the same group of patients who were treated only with intra-articular HA injections. The results of treatment by rinsing the joint cavities and HA viscosupplementation are presented in Table 5.

Gouveia et al. [41] demonstrated a strong correlation between the range of mandibular opening and satisfaction of patients with the quality of mastication. In our study, due to the complexity of mastication, the mobility of the mandible was evaluated by examination in three planes. HA injections improved the function of the mandible not only in terms of the mandibular opening—the amplitudes of protrusive and lateral movements also increased.

While the influence of HA viscosupplementation on the mandibular opening has been confirmed by numerous authors, data on the activity of the mandible in the transverse plane are very limited. These are presented by Chandrashekhar et al. [37], based on a study of 50 patients in whom they performed joint cavity rinsing with Ringer’s solution. Before treatment, the mean right lateral motion of the mandible was 7.2 mm. The maximum amplitude of the opposite direction was 7.6 mm on average. The mandibular mobility in patients treated by Chandrashekhar et al. [37] improved by 33% and 23%, taking into account right- and left-hand movements, respectively. In our study group, the improvement of mandibular function was 9% to the right and 11% to the left. Although scarce literature data do not allow us to formulate general conclusions, however, a clear difference in the effectiveness of joint lavage and HA viscosupplementation seems to be in favor of the former method. This would be in line with the analysis of the data already carried out on the increase in the amplitude of the mandibular opening.

The possibility of combining joint rinsing with HA viscosupplementation should also be considered. In a unique study by Gurung et al. [25], the highest efficacy in improving chewing function by using this combination of therapies was demonstrated. The amplitude of mandibular opening in the group of patients treated with a combination of joint rinsing and viscosupplementation was 13% higher than in the control group treated with joint rinsing alone. Moreover, the pain present during the protrusive and lateral movements of the mandible disappeared in all patients treated by both methods as early as 6 weeks of therapy. In the case of a group treated only with joint rinsing, the pain was still present in 20% of people after 12 weeks.

The influence of HA viscosupplementation on the degenerative changes of the articular fossa and head is also worth mentioning. In our own examination, radiological assessment of joint surfaces was performed on the basis of orthopantomograms and functional radiographs of temporomandibular joints. Degenerative changes on these surfaces were found in 8 patients before the treatment. Among them, remodeling was observed in only one patient who had previously been diagnosed with osteophytes. In the remaining 7 patients who were diagnosed with various resorption lesions before the treatment, no noticeable improvement in the condition of joint surfaces was observed. Similar observations were presented by Sun et al. [42] who injected HA into both compartments of the temporomandibular joints of 51 patients and evaluated the effects of the therapy using cone-beam computed tomography—no improvement in the condition of joint surfaces was found, as well as no slowdown in bone pathology progression.

Many authors have emphasized the significant influence of psychological components on the development of TMD. For example, in our study, we found a positive correlation between muscle and joint pains and stress (Pearson’s correlation coefficient *r* = 0.30), which indicates a tendency for pain to coexist with increased stress. Indirectly, this result may also prove the participation of psychological components in the process of initiation and evolution of TMD.

TMD is often discussed in the context of somatic diseases, but also stress and mental disorders. For example, Czerwińska-Niezabitowska and Kulesa-Mrowiecka [11] discussed the etiology of psychogenic defects of posture and educational therapy in the treatment of TMD. According to these authors, stress is a clear causative factor of TMD. Primary emotions such as anger or anger manifest themselves as increased tension of masticatory muscles innervated by the trigeminal nerve. The motor nucleus of this nerve is connected to the limbic system by gamma loops, which predisposes to teeth clenching and may explain such phenomena as clenching, bruxism, and other oral parafunctions. Among the patients with SLE dysfunction covered by this study, 88% of individuals suffered from exposure to severe and moderate stress. A relationship between stress and TMD can also be found in other authors’ publications. For example, Augusto et al. [43], in a study conducted on a group of 586 students of medical universities, showed a statistically significant relationship between TMD and parafunctions, stress, and mental illnesses. Similar observations were made by Ahuja et al. [44] who, on the basis of a study of a group of 450 people, found stress to be an important causative factor of TMD.

## 6. Conclusions

According to the results of our own research and the literature analysis, the high short-term effectiveness of HA viscosupplementation should be taken into consideration when treating pain and functional limitations related to TMD. Although it was most effective in reducing joint pain, good results were also observed for muscle pain. HA viscosupplementation positively influenced the mandibular opening pathway and amplitude. It also increased mandibular mobility in other directions. The verification of late treatment effects of hyaluronic acid viscosupplementation requires a continuation of the research.

## Figures and Tables

**Table 1:** Characteristics of the study group.

Age group	18–30 years old	8 patients (20%)
	30–44 years old	13 patients (33%)
	45–59 years old	14 patients (35%)
	60–69 years old	5 patients (13%)
Sex	females	36 patients (90%)
	males	4 patients (10%)
Duration of the ailment	≤6 months	11 patients (28%)
	> 6 ≤ 12 months	9 patients (23%)
	> 1 ≤ 3 years	12 patients (30%)
	>3 years	8 patients (20%)
Exposure to stress	no stress	2 patients (5%)
	minimal stress	3 patients (8%)
	moderate stress	9 patients (23%)
	severe stress	26 patients (65%)

**Table 2:** Qualification of the examined group according to the Schiffman et al. [8] classification.

Temporomandibular Joint Disorders
1. Joint pain
1.A. Arthralgia
1.B. Arthritis
2. Joint disorders
2.A. Disc disorders
2.A.1. Disc displacement with reduction—25 patients (63%)
2.A.2. Disc displacement with reduction with intermittent locking
2.A.3. Disc displacement without reduction with limited opening—2 patients (5%)
2.A.4. Disc displacement without reduction without limited opening—1 patient (3%)
2.B. Other hypomobility disorders
2.B.1. Adhesions / adherence
2.B.2. Ankylosis
2.B.2.(a) Fibrous
2.B.2.(b) Osseous
2.C. Hypermobility disorders
2.C.1. Dislocations
2.C.1.(a) Subluxation
2.C.1.(b) Luxation
3. Joint diseases
3.A. Degenerative joint disease
3.A.1. Osteoarthrosis—3 patients (8%)
3.A.2. Osteoarthritis—9 patients (23%)
3.B. Systemic arthritides
3.C. Condylysis / idiopathic condylar resorption
3.D. Osteochondritis dissecans
3.E. Osteonecrosis
3.F. Neoplasm
3.G. Synovial chondromatosis
4. Fractures
5. Congenital/developmental disorders
5.A. Aplasia
5.B. Hypoplasia
5.C. Hyperplasia

**Table 3:** Presence of muscular and articular pain in the study group before and after intra-articular HA injections.

Parameter	Value	Before the Injections	After Completion of the Injection Series
Muscle pain	present (+)	36 patients (90%)	14 patients (35%)
	absent (−)	4 patients (10%)	26 patients (65%)
Joint pain	present (+)	25 patients (62.5%)	3 patients (7.5%)
	absent (−)	15 patients (37.5%)	37 patients (92.5%)

**Table 4:** Mean extreme movements of the mandible in three planes.

	Opening	Protrusion	Lateral Movement—Right	Lateral Movement—Left
Initial value	40.1 mm SD = 8.0 mm	5.4 mm SD = 1.8 mm	7.8 mm SD = 2.4 mm	8.0 mm SD = 2.3 mm
Final value	44.6 mm SD = 6.0 mm	7.2 mm SD = 1.4 mm	8.5 mm SD = 2.1 mm	8.9 mm SD = 1.8 mm
Difference	4.5 mm SD = 4.2 mm	1.7 mm SD = 1.4 mm	0.7 mm SD = 1.3 mm	0.9 mm SD = 1.2 mm
Percentage difference	11%	31%	9%	11%
Correlation between the difference and the initial value	*r* = −0.68	*r* = −0.61	*r* = −0.49	*r* = −0.62

SD—standard deviation. Correlation expressed in Pearson’s correlation coefficient (*r*).

**Table 5:** Summary of the initial and final amplitudes of mandibular opening in other authors’ studies.

Authors (Date of Publication)	Therapy	Mean Initial Value (*x*)	Mean Final Value	Expected Final Value of HA Treatment Case (*y*)	Expected Final Value of AL Treatment Case (*y*)
Own test group	HA	40.1 mm	44.6 mm	not applicable	47.0 mm
Gurung et al. (2017) [25]	JL	37.2 mm	42.5 mm	41.0 mm	not applicable
Fonseca et al. (2018) [34]	HA	30.0 mm	37.0 mm	not applicable	41.0 mm
Neeli et al. (2010) [35]	JL	29.8 mm	41.9 mm	36.6 mm	not applicable
Malik and Shah (2014) [36]	JL	23.7 mm	41.0 mm	32.9 mm	not applicable
Chandrachekhar et al. (2015) [37]	JL	32.1 mm	46.6 mm	38.0 mm	not applicable
Leibur et al. (2015) [38]	JL	31.0 mm	44.0 mm	37.3 mm	not applicable
Jamot et al. (2017) [39]	JL	14.2 mm	27.6 mm	27.2 mm	not applicable
De Barros Melo et al. (2017) [40]	JL	46.0 mm	50.0 mm	46.3 mm	not applicable

The therapy column uses abbreviations: JL—joint lavage, HA—hyaluronic acid. The expected effect of HA viscosupplementation was calculated for a given test group on the basis of the formula *y* = *x* − 0.4 × *x* + 18.7 mm. The expected effect of arthroscopic lavage was calculated for a given study group on the basis of the formula *y* = *x* − 0.4 × *x* + 23.0 mm.
